# Avoiding Axillary Sentinel Lymph Node Biopsy after Neoadjuvant Systemic Therapy in Breast Cancer: Rationale for the Prospective, Multicentric EUBREAST-01 Trial

**DOI:** 10.3390/cancers12123698

**Published:** 2020-12-09

**Authors:** Toralf Reimer, Aenne Glass, Edoardo Botteri, Sibylle Loibl, Oreste D. Gentilini

**Affiliations:** 1 Department of Obstetrics and Gynecology, University of Rostock, 18055 Rostock, Germany; 2 Institute of Biostatistics, University of Rostock, 18055 Rostock, Germany; aenne.glass@uni-rostock.de; 3 Department of Research, Cancer Registry of Norway, 0304 Oslo, Norway; edoardo.botteri@kreftregisteret.no; 4 German Breast Group, 63236 Neu-Isenburg, Germany; sibylle.loibl@gbg.de; 5 Breast Surgery Unit, San Raffaele University and Research Hospital, 20132 Milan, Italy; gentilini.oreste@hsr.it

**Keywords:** breast cancer, de-escalation surgery, neoadjuvant therapy, sentinel lymph node biopsy

## Abstract

**Simple Summary:**

Improvements in systemic treatments for breast cancer have increased the rates of pathologic complete response (pCR) in patients receiving preoperative systemic therapy (PST), offering the opportunity to de-escalate, and perhaps eliminate, surgery in patients who have a pCR. We propose a clinical trial in which only patients with the highest likelihood of having a pCR after PST will be included and type of surgery will be defined according to the response to PST rather than on the classical T (for tumor size in the breast) and N (for axillary lymph node involvement) status at presentation. In the planned trial, axillary surgery will be eliminated completely (no axillary sentinel lymph node biopsy) for initially clinical node-negative patients with radiologic complete remission and a breast pCR as determined in the lumpectomy specimen.

**Abstract:**

Currently, axillary surgery for breast cancer is considered only as staging procedure, since the risk of developing metastasis depends on the biological behavior of the primary. The postsurgical therapy should be considered on the basis of biologic tumor characteristics rather than nodal involvement. Improvements in systemic treatments for breast cancer have increased the rates of pathologic complete response (pCR) in patients receiving neoadjuvant systemic therapy (NAST), offering the opportunity to de-escalate surgery in patients who have a pCR. European Breast Cancer Research Association of Surgical Trialists (EUBREAST)-01 is a clinical trial in which only patients with the highest likelihood of having a pCR after NAST (triple-negative or HER2-positive breast cancer) will be included and type of surgery will be defined according to the response to NAST rather than on the classical T (for tumor size in the breast) and N (for axillary lymph node involvement) status. In the discussed trial, axillary surgery will be eliminated completely (no axillary sentinel lymph node biopsy) for initially clinical node-negative (cN0) patients with radiologic complete remission and a breast pCR in the lumpectomy specimen. The trial design is a multicenter single-arm study with a limited number of patients (*n* = 267), which might give practice-changing results in a short period of time, sparing the time and the costs of a randomized comparison.

## 1. Introduction

The axillary lymph node dissection (ALND) with removal and histopathological examination of at least 10 nodes was an inherent part of surgical treatment of breast cancer for a considerable time. During the last two decades ALND was gradually replaced by sentinel lymph node biopsy (SLNB) in patients with clinically and sonographically unsuspicious lymph nodes [1,2,3]. As a consequence, the arm morbidity has reduced markedly [4]. Due to nationwide mammography screening in most industrial countries, a greater number of smaller tumors without axillary lymph node involvement is detected. More than 60% of all primary operable breast cancers do not have axillary lymph node metastases. This, in turn, means that even SLNB represents an overtreatment and is not advantageous for the majority of patients.

Neoadjuvant systemic therapy (NAST) serves two main goals. It provides effective systemic treatment (equivalent to adjuvant therapy) to prevent cancer recurrence, and allows de-escalating surgery for many women with larger tumors and/or axillary nodal involvement. Additionally, NAST permits assessment of tumor sensitivity to therapy in vivo leading to adjustment of post-neoadjuvant treatment based upon the pathological response to NAST. Patients showing an incomplete pathological response to NAST are candidates for capecitabine in triple-negative breast cancer (TNBC) [5] or for trastuzumab emtansine (T-DM1) in HER2-positive disease [6]. Currently, the use of NAST is the preferred initial treatment approach for stage II or III, HER2-positive or TNBC. In a woman who presents with a clinically negative axilla and who receives NAST, the St. Gallen Consensus Panel in 2017 strongly believed SLNB to be appropriate and favored it be carried out after NAST [7].

Non-invasive methods like ultrasound have gained importance in axillary lymph nodes staging. With improved insight into primary tumor biology and focus on residual disease after NAST, the axillary nodal status is still relevant for post-neoadjuvant treatment decisions. In cases with positive sentinel lymph nodes (SLN) the indication for completion ALND is clearly given in cases with macrometastasis. The St. Gallen Panel in 2017 was split on whether residual micrometastatic lymph node involvement warrented completion ALND after NAST [7]. 

The hypothesis of the ongoing European Breast Cancer Research Association of Surgical Trialists (EUBREAST)-01 (GBG104, NCT04101851) trial is to prove the oncological safety of omission of axillary SLNB after pathologic complete response (pCR) in the breast in response to NAST for TNBC and HER2-positive disease in initially clinical node-negative (cN0) patients.

EUBREAST-01 is an international prospective non-randomized, single-arm surgical trial designed by EUBREAST founding members. The University Medicine Rostock (UMR, Germany) will conduct the trial in collaboration with the German Breast Group (GBG Forschungs GmbH, Neu-Isenburg, Germany).

## 2. Evidence for Axillary Surgery and Lymph Node Involvement in Patients with Breast pCR after NAST 

### 2.1. Guidelines and Reviews

According to the 2014 American Society of Clinical Oncology (ASCO) SLNB guidelines, clinicians may offer cN0 women with operable breast cancer axillary SLNB either before or after planned NAST [8]. The updated German S3-guidelines recommend SLNB after NAST for patients with clinically and sonographically node-negative (cN0/iN0) pre-treatment status [9]. Completion ALND is a standard of care when nodal metastases are detected after NAST, however, the ongoing clinical trial ALLIANCE A011202 (NCT01901094) compares completion ALND versus axillary radiation in patients with a positive SLNB (ypN+(sn)) after NAST. 

The current data for axillary management after NAST, with a special focus on patients initially presenting as cN0, were summarized in a recent review [10]. The accuracy of SLNB after NAST in cN0 patients is similar to upfront SLNB. The long-term consequences of leaving non-sentinel lymph nodes with potentially chemoresistant disease in situ are still unknown, because the majority of SLNB studies after NAST required completion ALND to establish the accuracy of the procedure. There are data from the MD Anderson study, however, on 409 patients and SLNB alone (out of 575 patients initially cN0 with SLNB after NAST): the regional recurrence rate was 1.2% after 47 months follow-up [11].

This low regional recurrence rate is in concordance with the <2.5% at 10 years reported in the combined survival analysis of the NSABP B-18/B-27 trials for pre-entry cN0 patients treated with breast-conserving surgery (BCS) after NAST. Here, clinical nodal status before NAST, but not final pathologic nodal status, was an independent predictor for locoregional recurrence [12]. Improvements in systemic therapy for breast cancer have increased the rates of pathologic complete response (pCR) in patients receiving NAST, offering the opportunity to decrease, and perhaps eliminate, surgery in patients who have a pCR [13]. The concordance between axillary and breast pathologic response is different regarding cN0 and cN+ subgroups.

### 2.2. Retrospective Analyses Using Cancer Registry Data

A recent retrospective MD Anderson study showed that breast pCR after NAST correlated with nodal pCR after NAST. This study included 290 patients with triple-negative/HER2-positive breast cancer with T1-2 cN0/iN0 disease. Of the 116 patients (40.4%) who had a breast pCR, none (0.0%) had evidence of axillary lymph node metastases after NAST. Among 237 patients with biopsy-proven pre-treatment N1 disease, 89.6% of patients with a breast pCR had no evidence of axillary metastases after NAST, while 57.5% of patients without a breast pCR had residual axillary metastases [14]. Rates of breast and nodal pCR with NAST differ with tumor subtypes and are higher in TNBC or HER2-positive tumors when compared to hormone receptor-positive/HER2-negative disease [15]. A recently published retrospective analysis of the Netherlands Cancer Institute (*n* = 303 with initially cN0 status before NAST) confirmed the MD Anderson study [16]. In general, the SLNB-positive rate was low among TNBC (2.0%)/HER2-positive patients (0.0%) with a radiologic complete response (rCR) on breast magnetic resonance imaging (MRI) and was extremely low (0.0%) in cases with pCR in the breast. 

Barron et al. extended the retrospective evaluation of nodal positivity rates in cN0 patients with HER2-positive (*n* = 3062) and TNBC (*n* = 2315) with a breast pCR after NAST using the National Cancer Database [17]. In patients with cN0 HER2-positive disease or TNBC with breast pCR, the nodal positivity rate was 1.6% for both subtypes. Rates of ypN positivity were higher in patients with cN0 and residual disease in the breast after NAST (16.9% for HER2-positive and 12.6% for TNBC). Among patients with initially cN1 and HER2-positive disease, 43.3% achieved breast pCR with 12.4% of those being ypN positive. Corresponding data for cN1 TNBC were 37.3% for achieving breast pCR and 14.1% for being ypN positive.

Identical rates for ypN positivity in initially cN0 patients with breast pCR after NAST (1.6% for ER+/HER2+, 0.0% for ER−/HER2+, and 1.5% for TNBC subtype) were reported by Samiei et al. using data from the Netherlands Cancer Registry [18]. The odds of ypN0 were decreased in cases of clinical T3 stage (OR 0.59), cN1 (OR 0.03), and ER-positive/HER2-negative subtype (OR 0.30). Taken together, in patients with cN0 HER2-positive disease or TNBC with breast pCR, the nodal positivity rate was less than 2%, which supports consideration of omission of axillary surgery in this subset of patients.

## 3. Discussion of the Study Design

EUBREAST-01 is a prospective non-randomized, single-arm surgical trial. A randomized design is not useful due to expected extremely low axillary recurrence rates after 3 and 5 years for the experimental arm. In case of a two-arm randomized setting, the risk for underpowered testing because of a low number of events will be considerably high.

The trial is designed as an uncontrolled, single-arm study comparable with the Adjuvant Paclitaxel and Trastuzumab (APT) trial for node-negative, HER2-positive breast cancer [19]. This type of study design is very helpful to obtain robust and reliable results in a short period of time saving both time and resources which are needed to conduct a randomized trial. The duration of recruitment will be 2 years among 30 German, 10–15 Italian, 3 Swedish, 3 Spanish, and 3 Austrian study centres. The total number of patients to be recruited will be 267. Ethics statement: The study was designed in accordance with the Declaration of Helsinki, and the protocol was approved by the Ethics Committee at the University of Rostock, Germany (A2019-0092). 

### 3.1. Time Lines

- First patient in: Q1/2021
- Last patient in: Q4/2022
- First analysis: Q4/2025
- Final analysis: Q4/2027

### 3.2. Current Sstandard Treatment

Since 2005, axillary dissection with removal of at least 10 lymph nodes (ALND) was gradually replaced by SLNB as a staging procedure in Germany [20,21]. Since then, the indication for SLNB rapidly expanded. According to current German AGO guidelines version 2020.1 [22], all patients with initially cN0 status should undergo axillary SLNB after NAST. In case of ypN0(sn) finding no completion ALND must be performed; axillary SLNB alone has the “++” recommendation level. In contrast, SLNBs with ypN1(sn) results require completion ALND with respect to axillary tumor load: “+” recommendation for pN1mi(sn); “++” recommendation for pN1a(sn). Currently, axillary radiotherapy (ART) is an alternative option with “+/−” as the recommended level. Multifocal or multicentric disease is not a contraindication for undergoing SLNB.

### 3.3. Experimental Study Arm

After rCR at the end of NAST all patients will be treated with lumpectomy (BCS) alone without any axillary surgery. Approximately 80% of these patients will be assigned to the single study arm (no axillary SLNB) due to breast pCR (ypT0) at the final pathology.

**Figure 1:**
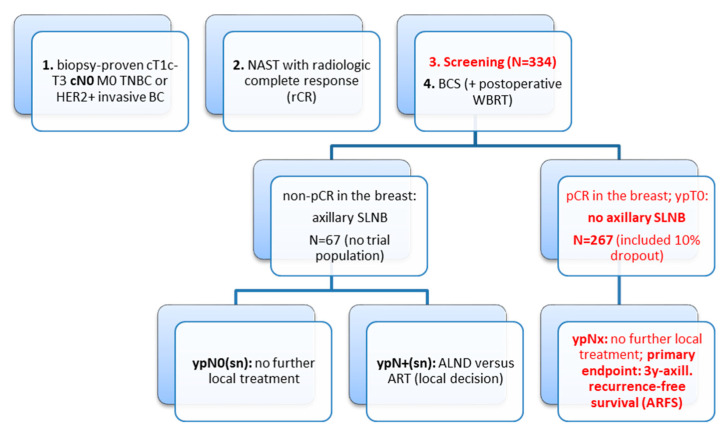
Flow chart of the EUBREAST-01 (GBG104) trial according protocol amendment #1. Assuming a 10% dropout rate for per-protocol analysis the required sample size for experimental single-arm is *n* = 267 (which is the expected 80% proportion of the screening population). TNBC: triple-negative breast cancer (ER-/PgR-/HER2-); BC: breast cancer; NAST: neoadjuvant systemic therapy; BCS: breast-conserving surgery; WBRT: whole-breast radiotherapy; SLNB: sentinel lymph node biopsy; pCR: pathologic complete response; cALND: completion axillary lymph node dissection; ART: axillary radiotherapy.

In patients with initially cN0 status and HER2-positive disease or TNBC with breast pCR, the nodal positivity rate after NAST is less than 2% using retrospective data [17,18]. In contrast, the ypN positivity rate will be increased to ≥10% in cN0 cases without breast pCR. The flow chart of the EUBREAST-01 trial, according protocol amendment #1 is shown in Figure 1; a summary of retrospective Cancer Registry based data is listed in Table 1.

### 3.4. Selection of Study Population

The main inclusion criteria are clinically (cN0) and sonographically (iN0) negative axillary status before NAST and planned BCS with postoperative radiotherapy. In cases with cN0 and iN+, a negative core biopsy or fine needle aspiration (FNA) of the sonographically suspected lymph node is required before NAST. The study population is restricted to patients with rCR at the end of NAST and proven breast pCR at the final pathology of the BCS. 

The population of the EUBREAST-01 trial will include patients (≥18 years) of two intrinsic subtypes described for breast carcinoma. The background for the decision to recruit only patients with HER2-positive disease or TNBC is supported by the following reasons:

1. NAST is the standard approach for these two subtypes, at least for stage II and III [7].
2. The highest rates of breast pCR rates were seen in these two subtypes [17].
3. The highest rates of axillary nodal pCR (ypN0) rates were described for these two subtypes [10]. Accordingly, the lowest rates for ypN positivity after NAST were observed in these two subtypes [18].

## 4. Primary Endpoint

The 3-year rate of axillary recurrence-free survival (ARFS), defined as no tumor recurrence in lymph nodes in the ipsilateral axilla, infra-/supraclavicular fossa, or interpectoral area. Recurrencies must be confirmed with core biopsy, FNA, or local excision (open biopsy).

## 5. Secondary Endpoints

The following secondary objectives are defined:

- 5-year invasive disease-free survival
- 5-year overall survival
- 5-year locoregional disease-free survival
- 5-year distant disease-free survival
- 5-year ARFS
- 5-year ipsilateral axillary recurrence rate
- Diagnostic accuracy of imaging methods for pathologic complete response (breast pCR) after NAST. Regarding the secondary outcome of diagnostic accuracy of imaging methods, the potential value when using optional breast MRI will be evaluated.

No quality-of-life (QoL) parameters were considered as secondary endpoints due to the single-arm, uncontrolled trial design (no comparison between treatment arms available). Statistical analyses will be conducted after a follow-up of 3 years for the primary endpoint and after 5 years follow-up for the secondary endpoints. No interim analysis is planned due to expected low event rates. Patients will be assessed for disease recurrence according to standard national clinical practice over a period of at least 5 years. The longest follow-up will be 7 years. History and physical examination will be performed every 6 months for the first 36 months and yearly thereafter. Annual mammography and sonography will be required; other testing will be based on symptoms and investigator preference.

## 6. Inclusion Criteria

- Written informed consent prior to BCS
- Histologically confirmed unilateral primary invasive carcinoma of the breast (core biopsy). Multifocal or multicentric tumors are allowed if BCS is planned.
- Age at diagnosis at least 18 years
- Imaging techniques with estimated tumor stage between cT1c-T3 prior to NAST
- Triple-negative or HER2-positive invasive breast cancer
- Clinically and sonographically tumor-free axilla prior to core biopsy (cN0/iN0)
- In cases with cN0 and iN+, a negative core biopsy or FNA of the sonographically suspected lymph node is required
- No evidence for distant metastasis (M0)
- standard NAST with radiologic complete response (rCR)
- Planned BCS with postoperative external whole-breast irradiation (conventional fractionation or hypofractionation)

## 7. Exclusion Criteria

- History of malignancy within the last 5 years, except curatively treated basalioma of the skin and carcinoma in situ of the cervix
- Time since last cycle of NAST > 3 months (optimal <1 month)
- Histologically non-invasive breast carcinoma
- Hormone receptor-positive/HER2-negative disease (triple-positive tumors are allowed)
- cT4 or iT4 tumors
- Pregnant or lactating patients
- No radiological complete response at the end of NAST
- Planned total mastectomy after NAST
- Planned intraoperative radiotherapy (e.g., Intrabeam) or postoperative partial breast irradiation (e.g., multicatheter technique) alone; both procedures are allowed as boost techniques
- Male patients

## 8. Imaging of Response to Standard NAST

Several imaging techniques are available to predict the effect of NAST among patients with breast cancer. In many recent trials, physical examination, mammography, ultrasound have still been used as the primary diagnostic tools for the evaluation of therapy. Digital breast tomosynthesis may be utilized as part of the diagnostic evaluation because this method has the potential to improve the measurement accuracy due to reducing the masking effect from adjacent normal tissue. Additional challenges with mammography include the presence of microcalcifications, which do not correlate with presence of a viable tumor [23]. According to the revised Response Evaluation Criteria in Solid Tumors (RECIST) guideline, the value of ultrasound in clinical trials is questionable for the measurement of tumor regression because the examination is subjective and operator dependent [24]. The best conventional imaging method for predicting pCR appears to be the combination of mammography with ultrasound (80% likelihood when findings of both modalities are negative) [23].

Some studies have shown that MRI can determine the response to NAST more accurately than mammography, ultrasound, and clinical examination. Yuan et al. [25] performed a meta-analysis of MRI value in the prediction of pCR after NAST. This meta-analysis (*n* = 1212) has shown that contrast-enhanced MRI has a high specificity (90.7%) and relative low sensitivity (63.1%) in predicting pCR after NAST. In other studies, it was observed that MRI was able to predict pCR more accurately in patients with HER2-positive disease or TNBC [26]. Utilization of breast MRI for evaluation of the response to NAST is included in the EUBREAST-01 protocol as an optional tool (strongly recommended if locally available and reimbursed). Despite inclusion in several clinical guidelines (e.g., National Comprehensive Cancer Network (NCCN): “May be helpful for breast cancer evaluation before and after NAST…” [27]), preoperative breast MRI is not universally available. 

Prior to initiation of NAST, diagnostic imaging should be performed for both breasts (plus axillary lymph nodes) with routine staging procedures to rule out primary metastatic disease. Clip-placement in the breast tumor lesion is strongly recommended at the beginning of NAST. The same imaging modalities and protocols should be performed after completion of NAST for measurement of the response. Currently, the use of functional imaging (e.g., 18F-fluorodeoxyglucose positron emission tomography) as an alternative assessment method is not supported by the EUBREAST-01 investigators due to insufficient standardization and widespread availability. 

There are currently no standards for reporting imaging assessment of the tumor response to NAST. The types of imaging responses are categorized as complete response, partial response, stable disease, or progressive disease [23]. The rCR is defined as complete resolution of original imaging finding; in case of mammography with or without microcalcifications. Patients without rCR will be excluded before the EUBREAST-01 screening phase. Unfortunately, we expect that a small cohort of patients with no rCR but pCR in the breast will be excluded due to restriction for patients with rCR after NAST. EUBREAST-01 will not provide documentation for these excluded patients during pre-surgical screening, so we cannot answer the question regarding percentage of patients with incomplete radiological response (e.g., caused by scarring or fibrosis) and pCR in the breast. 

## 9. Screening Phase

All initially cN0 patients with a rCR after standard NAST for HER2-positive disease or TNBC are candidates for the planned trial. HER2 status is considered positive if an immunohistochemistry (IHC) score of 3+ is recorded, or if there is positive gene amplification using in situ hybridization testing. TNBC will be diagnosed as an IHC-defined subtype: ER- and progesterone receptor (PgR)-negative/HER2-negative. The rCR will be determined by mammography and ultrasound of the breast plus axilla at the end of NAST. MRI is an option, but not mandatory for evaluation of rCR. 

## 10. Surgical Therapy

The expected rate of breast pCR (ypT0) in cases with rCR after standard NAST is approximately 80% [23]. Breast pCR is defined as no invasive or in situ cancer in the breast (ypT0) based on the German Breast Group (GBG) definition of pCR in the neoadjuvant setting [28]. All patients with confirmed breast pCR after lumpectomy (BCS) will be selected for the single study arm (no axillary therapy) leading to omission of any axillary treatment (axillary SLNB, ALND, and/or axillary radiotherapy). These patients will thus be finally staged as ypNx. 

Patients with non-pCR in the breast will be treated with axillary SLNB in a second procedure in concordance with current guidelines outside from EUBREAST-01 protocol. That means, in case of a tumor-free SLNB (ypN0(sn)), no completion ALND is performed. If micro- or macrometastases are found in the SLNB (ypN+(sn)), completion ALND and/or axillary radiotherapy is mandatory according to local decision. A patients’ advocate was involved during the development of the trial design using the EUBREAST network. There were no major concerns regarding the two-stage SLNB procedure in case of non-pCR in the breast.

According to current guidelines, axillary SLNB is only indicated in patients with histologically proven invasive breast cancer and clinically (cN0) and sonographically (iN0) insuspect lymph nodes. The initial diagnostic work-up must include clinical examinations of both axillary regions and routine axillary ultrasound before biopsy. Suspected palpable axillary lymph nodes are an exclusion criterion for the study. In cases with cN0 and iN+, a negative core biopsy or FNA of the sonographically suspected lymph node is required before NAST. Currently, there are no clear criteria defined for simple but reproducible and validated sonographic criteria to categorize patients as iN0 correctly in the preoperative setting. Local investigators should be focused on cortical changes (possible cut-off 2.5 mm) and the absence of a fatty hilum to diagnose metastatic lymph nodes by axillary ultrasound [29].

The breast carcinoma must be confirmed by core biopsy before NAST. Lumpectomy after wire-localization of the clip-marked tumor bed will be performed as standard procedure according to local site guidelines. An intraoperative radiography of the removed surgical specimen will be recommended by the EUBREAST-01 investigators. An intraoperative frozen section procedure should be avoided for investigation of breast tissue. A clip-placement to the area of removed tumor bed is recommended at the end of lumpectomy for optimal planning of postoperative boost radiation. Final allocation to the experimental arm will be completed after receiving the final pathologic report. Therefore, all axillary SLNBs in case of non-pCR in the breast are planned as two-stage procedures. 

The published recommendations for standardized evaluation of the post-NAST surgical specimen in breast cancer neoadjuvant trials promote accuracy and reproducibility of the response assessment across institutions [30].

## 11. Postoperative Radiotherapy

Adjuvant radiotherapy should be applied according to the current AGO guidelines [22], updated German S3-guidelines (December 2017), and DEGRO practical guidelines [31]. All study patients must receive postoperative CT-based whole-breast irradiation (WBI) with three-dimensional conformal radiation therapy (3DCRT) to the remaining breast (50 Gy in 25 fractions or 50.4 Gy in 28 fractions). In addition, the hypofractionated regimen with a single dose of 2.66 Gy in 15 fractions according to the START B trial [32] is a valid option. The extension of radiation fields to high tangents (target contoured level I and middle to upper-level II axillary lymph nodes) or comprehensive fields (high tangents plus supraclavicular) is not recommended. Hypofractionated WBI delivered in 1 week according to the FAST-Forward trial [33] is not an option before implementation in national guidelines.

A boost to the tumor bed is indicated for all patients because HER2-positive disease and TNBC are considered as high-risk subtypes. The recommended dose for the tumor bed boost is (10-)16 Gy. The application of the simultaneously integrated boost (SIB) technique is an option during normofractionated WBI, but is not allowed during hypofractionated WBI schedules [34]. In selected cases, modern techniques like intensity-modulated radiotherapy (IMRT), volumetric intensity-modulated arc therapy (VMAT), and radiotherapy in deep inspiration breath hold are allowed as options for postoperative radiation [9].

## 12. Pre- and Postoperative Systemic Therapy 

Pre- and postoperative systemic treatment should be based on local multidisciplinary tumor board recommendations according to the current AGO breast guidelines [22].

- Hormone-sensitive (triple-positive) disease: patients should receive postoperative endocrine treatment according to current standard recommendations.
- All patients should receive neoadjuvant chemotherapy according to the current standard recommendations. Currently, postoperative chemotherapy is not indicated for patients with breast pCR after NAST.
- HER2-positive disease: all patients should receive anti-HER2-treatment according to current standard recommendations as neoadjuvant and post-neoadjuvant therapy; including routine cardiac assessment.
- Novel therapeutic modalities: PARP-inhibitors or immune checkpoint-inhibitors are allowed as treatment in clinical trials when ypNx status is not an exclusion criterion. If approved for the neoadjuvant or post-neoadjuvant setting, PARP-inhibitors or immune checkpoint-inhibitors are allowed as treatment according to standard recommendations.
- Bone modifying agents (bisphosphonates, denosumab) are allowed according to current treatment guidelines.

## 13. Sample Size Determination

The following assumptions are made for sample size calculation:

1. We expect to observe a 3-year ARFS of at least 98.5% [11,35]. This 3-year ARFS of ≥98.5% is estimated due to reported ypN+ rates of less than 2% for cN0 patients with breast pCR after NAST (Table 1) and according to published low axillary recurrence rates after NAST and SLNB alone (Table 2).

2. We consider a 3-year ARFS ≤ 96% to be unacceptable in this patient population. A 3-year ARFS ≥ 96.5% and <98.5% is still tolerated, because such a decrease (up to two percentage points) compared to our boundary of 98.5% does not indicate a clinical benefit of routine axillary SLNB in initially node-negative patients [38].

In almost all trials, completion ALND was performed to evaluate false-negative rates when SLNs were negative for metastasis; therefore, long-term follow-up data regarding axillary recurrence after SLNB without ALND are limited in the neoadjuvant setting. 

The assumption for acceptable 3-year ARFS ≥98.5% in the experimental arm is based on previous study findings, as discussed in Table 1; Table 2. Reported 3-year axillary recurrence rates of <1.5% were seen in trials including initially cN0 patients plus axillary intervention after NAST.

3. Exponentially distributed time to event (axillary recurrence), since the axillary recurrence seems to be an early event [35,39].

4. Exponentially distributed time to dropout.

5. The overall error rate of a false positive outcome (α) is set to 0.05. 

6. The error rate for a false negative outcome (β) is set to 0.05, i.e., the expected power of the trial is set to 95% to detect a difference of clinical interest (from 96% to 98.5%).

7. The accrual period during which patients enter the study is 24 months.

8. The follow-up period from the end of accrual until the analysis of data is 36 months.

9. The assumed 3-year dropout rate is 10%.

10. A two-sided test is used (H0: ARFS = 96 vs. HA: ARFS ≠ 96).

11. Subgroup analysis according to the following criterion:

Receptor status (HER2-positive disease vs. TNBC) 

We need to enroll 267 patients in order to have a well-powered study (power of 95%) with a type I error rate of 0.05, and including a 10% dropout rate for per-protocol analysis. Finally, we need to screen a total of 334 cases (hereof 267 with breast pCR, which amounts to an expected proportion of breast pCR of 80%).

## 14. Statistical Analyses

### 14.1. Evaluation of Primary Endpoint

Per-protocol and intention-to-treat (ITT) analyses will be conducted for all patients. The analysis regarding 3-year ARFS is planned after a follow-up of 3 years for the last enrolled patient, so that all patients are followed at least 3 years when analyzing for the primary outcome. The curve will be estimated using the Kaplan–Meier method, based on the per-protocol population, and stratified subgroups (HER2-positive disease versus TNBC) will be compared with a two-sided log–rank test. The Cox-proportional hazards model will be used in a univariate approach to select potential factors influencing the hazard, with the aim to include them in a subsequently applied multivariate approach. Here, hazard ratios will be adjusted for the selected factors.

### 14.2. Evaluation of Secondary Endpoints

Secondary outcomes (5-year rates of ARFS, invasive disease-free survival, overall survival, locoregional disease-free survival, distant disease-free survival) are defined as the time period between BCS and the first event and will be analyzed after a median follow-up of 5 years by referring to data from GBG patient’s registry. The 5-year axillary recurrence rate (with the corresponding 95% confidence interval (CI) will be reported after a 5-year follow-up documentation for each patient. Finally, the diagnostic accuracy rate of breast imaging methods (including optional MRI) for predicting breast pCR after standard NAST will be reported.

## 15. Conclusions

Three scenarios of results can be envisaged which will lead to the following conclusions:

- The experimental arm (no axillary SLNB) shows a high 3-year ARFS (≥98.5%). Omitting the axillary SLNB according to the inclusion criteria would be then considered as a new standard option for BCS of patients with neoadjuvant treated, primary breast cancer.
- The experimental arm (no axillary SLNB) shows an unacceptable 3-year ARFS rate of ≤96%. In this setting the current guidelines for SLNB are confirmed.
- The experimental arm (no axillary SLNB) shows an intermediate 3-year ARFS rate (96.1–98.4%). No final conclusion for routine clinical practice can be given; the conduction of a randomized clinical trial must be discussed.

## Figures and Tables

**Table 1:** List of trials with axillary interventions after neoadjuvant systemic therapy (NAST) reporting outcomes regarding ypN+ rate with respect to initially clinical nodal status and breast pCR.

Study for ypN+ Rate in cN0 Patients with Breast pCR (N) after NAST	ER+/HER2−	HER2+	TNBC
Barron et al. [17] *n* = 5377	n.d.	1.6%	1.6%
Samiei et al. [18] *n* = 986	6.7%	ER+/HER2+: 1.6% ER−/HER2+: 0.0%	1.5%
Tadros et al. [14] *n* = 116	n.d.	0.0%	0.0%
Van der Noordaa et al. [16] *n* = 89	0.0%	0.0%	0.0%

**Table 2:** Previous findings in neoadjuvant breast cancer trials with initially cN0 patients and axillary surgery after NAST with respect to axillary recurrence rates.

Study Characteristics (cN0; SLNB Alone after NAST)	Follow-Up	Axillary Recurrence Rate
Nogi et al. [36] *n* = 183	51.1 months	0.0%
Galimberti et al. [37] *n* = 396	61 months	0.0%
Hunt et al. [11] *n* = 575 (*n* = 491 SLNB alone)	47 months	1.2%
Mamounas et al. [12] *n* = 1384 (ALND or SLNB)	10 years	0.5–2.3%
